# The experience of health and welfare workers in identifying and responding to domestic abuse among military personnel in the UK

**DOI:** 10.1186/s12913-020-05672-x

**Published:** 2020-10-15

**Authors:** Katherine Sparrow, Filipa Alves-Costa, Ana Alves, Neil Greenberg, Louise M. Howard, Nicola T. Fear, Deirdre MacManus

**Affiliations:** 1 Forensic and Neurodevelopmental Science Department, King’s College London, Institute of Psychiatry, Psychology and Neuroscience, 16 De Crespigny Park, London, SE5 8AF UK; 2 Barnet Enfield and Haringey Mental Health NHS Trust, London, UK; 3 King’s Centre for Military Health Research (KCMHR) Academic Department of Military Mental Health (ADMMH), King’s College London, London, UK; 4 Section of Women’s Mental Health, King’s College London, Institute of Psychiatry, Psychology and Neuroscience, London, UK

**Keywords:** Military, Veterans, Domestic abuse, Intimate partner violence, Healthcare services, Welfare services, Support services

## Abstract

**Background:**

Awareness of domestic violence and abuse (DVA) as a problem among military personnel (serving and veterans) has grown in recent years, and there is a need for research to inform improvements in the identification of and response to DVA in this population. This study aimed to explore the experience of health and welfare professionals in identifying and responding to DVA among the UK military population (serving personnel and veterans).

**Methods:**

Thirty-five semi-structured telephone interviews were conducted with health and welfare staff who work with serving UK military personnel and veterans. Interviews were analysed using thematic analysis.

**Results:**

Three superordinate themes were identified: i) patterns of DVA observed by health and welfare workers (perceived gender differences in DVA experiences and role of mental health and alcohol); (ii) barriers to identification of and response to DVA (attitudinal/knowledge-based barriers and practical barriers), and iii) resource issues (training needs and access to services). Participants discussed how factors such as a culture of hypermasculinity, under-reporting of DVA, the perception of DVA as a “private matter” among military personnel, and lack of knowledge and awareness of emotional abuse and coercive controlling behaviour as abuse constitute barriers to identification and management of DVA. Healthcare providers highlighted the need for more integrated working between civilian and military services, to increase access to support and provide effective care to both victims and perpetrators. Furthermore, healthcare and welfare staff reflected on their training needs in the screening and management of DVA to improve practice.

**Conclusions:**

There is a need for increased awareness of DVA, particularly of non-physical forms of abuse, and of male victimisation in the military. Standardised protocols for DVA management and systematic training are required to promote a consistent and appropriate response to DVA. There is a particular training need among healthcare and first-line welfare staff, who are largely relied upon to identify cases of DVA in the military. Employing DVA advocates within military and civilian healthcare settings may be useful in improving DVA awareness, management and access to specialist support.

## Background

Over the years a growing number of studies have suggested that DVA experiences, both perpetration and victimisation, may be more prevalent among military compared to civilian populations [1–5] and more severe [6]. Data from ongoing UK research comparing DVA in the UK military to the UK civilian population is awaited. The UK Ministry of Defence have stated that they presume that DVA is at least as prevalent in the military [7]. They acknowledge factors related to military service, which may increase the likelihood of DVA (e.g., experiences of military personnel) or impact on its reporting or management (e.g., dependence on perpetrator for financial support, perception that military will favour the perpetrator and not support survivors) [7].

There is a body of research literature, which supports the assertion that military couples can be exposed to unique stressors, which can negatively impact relationships and potentially increase the risk of DVA [8]. These stressors may be related to occupational specific factors such as deployment [9–11]. Indeed, higher levels of combat exposure have been shown to be associated with significantly higher prevalence of DVA [1–3, 12, 13]. Mental health difficulties (for some deployment-related), such as post-traumatic stress disorder (PTSD) and alcohol misuse, have been reported to be higher among UK military personnel compared to the general population [14, 15], with strong evidence to suggest that the two are co-occurring in UK military populations [16], and such disorders have been found to predict higher levels of DVA perpetration [10]. Additionally, alcohol misuse has been found to significantly moderate the association between PTSD and ‘past year’ partner violence with a positive correlation between high levels of alcohol misuse, PTSD symptoms and intimate partner violence [17]. It is also important to note that mental health difficulties can render a person to be more vulnerable to DVA victimization [18].

Some research [19–21] suggests a relationship between DVA in military couples and reintegration into family life following separations, for example following deployment, and following transition back into civilian life. Indeed reintegration periods can impact the relationship negatively, increase relationship dissatisfaction and increase perpetration of abusive behaviours towards partners [22, 23]. Relationship dissatisfaction has been found to be independently associated with both IPV perpetration and victimization [24, 25].

Disclosure and identification of DVA is problematic and one of the major barriers to management and appropriate care provision. Military couples might face additional barriers, such as perceiving the military-based support as problematic, have fears about the impact of help-seeking on their own or partner’s career, view help-seeking as a weakness, have concerns about lack of confidentiality within the military community [21], and perceive limited access to independent support services [26].

Research on effective practices in the identification of and response to DVA in the military in the UK is lacking. Whilst there has been debate over the implementation of routine screening for DVA in civilian healthcare settings, with some studies not finding sufficient evidence to support formal screening practices [27–30], the UK National Institute for Health and Clinical Excellence (NICE) issued quality standards for the management of Domestic Abuse by health and social care practitioners and service providers [31], which stipulated that practitioners should be able to recognise indicators of possible domestic violence and abuse and respond appropriately. They also stated that services should ensure practitioners are trained to do so. Some research conducted with military personnel and veterans have highlighted that screening for DVA can create opportunities for identification and management of DVA, increase opportunity for comprehensive assessments and access to IPV-related services for victims/survivors of DVA [32–37] and perpetrators of DVA [38].

A recent study highlighted the appropriateness of considering routine screening for DVA in military settings (military personnel and veterans) as it led to a better response (i.e., triage, signposting and appropriate intervention [39]. Nevertheless, some challenges were noted, such as time and resource constraints, competing responsibilities of professionals, lack of formal policies and guidelines on DVA screening and response procedures, as well as lack of appropriate training and discomfort inquiring. Veteran healthcare providers have also reported concern about lack of resources to respond to partner violence in military populations [40].

The international literature to date highlights the need for military specific strategies for the management of domestic abuse. Care providers from military welfare agencies in the UK have noted that service members require a unique approach as aggression is an inherent part of military training and acknowledged the potential impact of trauma, transience and deployment, and how it interacts with DVA within military populations [21]. In their first DVA strategy published in 2018, the UK Ministry of Defence outlined key areas for action (e.g., awareness raising, specialist training and appropriate policies, partnerships with external organisations), and, given the current lack of research in this area, highlighted a need for research to support and guide its further development.

There is an urgent need for research into the response to and management of DVA among UK military communities in order to guide the development of support services. This study aimed to explore healthcare and welfare professionals’ awareness of DVA, and their experiences of identifying and responding to DVA among serving personnel, veterans and their families in the UK.

## Methods

### Paradigmatic and ethical underpinnings

This qualitative research was undertaken as part of a wider NIHR funded mixed methods study into Domestic Abuse in the UK military, which aimed to investigate: (i) prevalence and risk factors of DVA; (ii) the impact of military life on relationship conflict and IPV and facilitators of and barriers to help-seeking; and (iii) attitudes and response of the UK military to DVA. This study was designed to contribute to the investigation of the latter. Pragmatism was adopted as the philosophical model. Pragmatism is a more flexible paradigm when compared with philosophies such as (post) positivism, interpretivism or constructivism which tend to describe the reality by purely considering quantitative or qualitative stands, respectively [41, 42]. On the contrary, a pragmatism paradigm advocates the use of mixed methods in order to build and interpret knowledge. This study was reviewed and approved by King’s College Research Ethics Committee (LRS127 15/16–3607).

### Patient and public involvement, consultation and reflexivity

Following a patient and public involvement (PPI) process allowed appraisal of our topic guide and semi-structured interview and adjustments were made accordingly. This included changes to terminology and phrasing of questions as well as length of the interview. The semi-structured interview was piloted with volunteers before data collection began. Findings from the current study (and wider project) are being disseminated among all of the participants. A PPI event was organised and attendees included representatives from the UK military (two-tier welfare support system), health and welfare practitioners and survivors of DVA. This allowed these findings to be presented, and validated.

Throughout the study, the research team has received consultation from senior authors, external researchers and practitioners. Meetings offered the opportunity for them to reflect on all steps of the study. This facilitated procedural decisions and detailed discussion of data generation and management in a transparent and explicit manner (ensuring trustworthiness of the data). Furthermore, the research team acknowledge that the interviewers’ backgrounds (white, middle-class, non-military females) could have impacted on the interviewees’ performances (e.g., social desirability bias). However, frequent meetings with external consultants were used to support our reflexivity (reflection on the influence of the researcher on the research), decreasing the possibility for bias (credibility). Thus, the data gathered might fit into similar contexts outside the study situation (applicability).

### Setting

The UK military has a two-tier welfare support system. The first point of contact is first line welfare support provided by Unit Welfare Officers (in the Army), Divisional Officers (in the Navy) and Personal Support Flight staff (in the Royal Air Force - RAF). Such personnel provide initial triage and signposting. Second line welfare services deliver confidential, specialist support, provided by the Army Welfare Service (AWS), Royal Navy Royal Marines (RNRM) Welfare and Soldiers, Sailors, Airmen and Families Association (SSAFA) for the RAF. Many of those working in second line welfare services are civilians. Healthcare is provided by a range of professionals (general practitioners, nurses, mental health professionals) working within the Defence Medical Services. For military personnel who have left service, their needs are met by community social and health care services.

### Participant selection and recruitment

A purposive sample was recruited to ensure diversity in gender, service, rank and/or professional role (Table 1). Potential participants were identified by approaching appropriate gatekeepers for the health and welfare services within/out the military. Participant information was provided, and volunteers invited to make contact. Prior to involvement in the study participants received full information about the research and provided written and verbal consent to participate. All participants received £25 to thank them for their time.

**Table 1:** Participant demographics

Total (*n* = 35)		
**Gender**	Male	12
	Female	23
**Military population to which the participant provides support**	Army	15
	RAF	9
	Navy	7
	Veterans	4
**Military service status**	Military **(M)**	19
	Civilian **(C)**	16
**Occupational role**	Leadership **(L)**	6
	Welfare/Social Worker **(WW)**	15
	Healthcare –GP **(H)**	4
	Healthcare – mental health worker (psychiatrist/psychologist/mental health nurse) **(H)**	10
**Geographical location**	England	28
	Northern Ireland/Scotland	5
	International	2

### Data collection

A semi-structured interview schedule was developed through consultation with experts in military health, mental health, DVA research, and those with experience providing support to military families and/or those experiencing domestic abuse, and user/carer representatives from military and veteran support services. The topic guide covered three main areas: i) participants’ awareness and experience of DVA in the military, ii) participants’ experiences of identifying DVA and (iii) participants experiences of managing DVA among military personnel. For instance, the research team asked: *Based on experience in your role, how would you describe the DVA reported by military personnel/ veterans/their partners*? *I was wondering if you have come across any barriers that you feel might prevent military personal/veterans/their partners from reporting domestic abuse? Have you experienced any difficulties in responding to/managing cases of DVA?.*

The Semi-structured telephone interviews were conducted by two experienced researchers (authors KS and DM) between October 2016 and March 2017. Following participants’ consent, interviews were digitally recorded and transcribed verbatim ready for coding. Each interview lasted between 40 and 60 min.

### Data analysis

Interviews were analysed using an inductive *Thematic Analysis* method [43, 44]. All the analyses were supported through the use of QSR NVivo11 software. The qualitative data analyses followed guidelines in the literature. An initial coding frame was developed by two researchers (KS and DM) based on the interview topic guide themes and simultaneous coding of the first five interview transcripts. Subsequently, overall codes were generated and revisited several times. Finally, an independent moderator (FAC) validated the coding system, including reviewing initial themes and subthemes, and amendments were made. When theoretical saturation was achieved, the recruitment ended. The research team included a second party conducting coding of transcripts to ensure data saturation has been reached [45]. The code saturation was reached to the point at which no additional data were identified and the codebook stabilized [46].

The suitability of the coding frame was assessed through progressive iterations and discussions within the research team. The systematic and transparent method of iterative categorization was followed when coding and preparing the qualitative data for analysis.

## Results

A total of 35 participants (12 men and 23 women) who work with UK serving military personnel and veterans took part in the study. Over half were currently serving in the military. Most worked as welfare/social workers or healthcare/mental health workers (psychiatrist/psychologist/mental health nurse) in England (Table 1). A minority were in leadership roles.

Three overarching themes were identified from the thematic analysis, as follows:

i) Patterns of DVA observed by health and welfare workers (subthemes: perceived gender differences in DVA experiences and role of mental health and alcohol);
ii) Barriers to identification and management of DVA (subthemes: attitudinal/knowledge-based barriers and practical barriers); and
iii) Resource issues (subthemes: training needs and access to services).

**Fig. 1:**
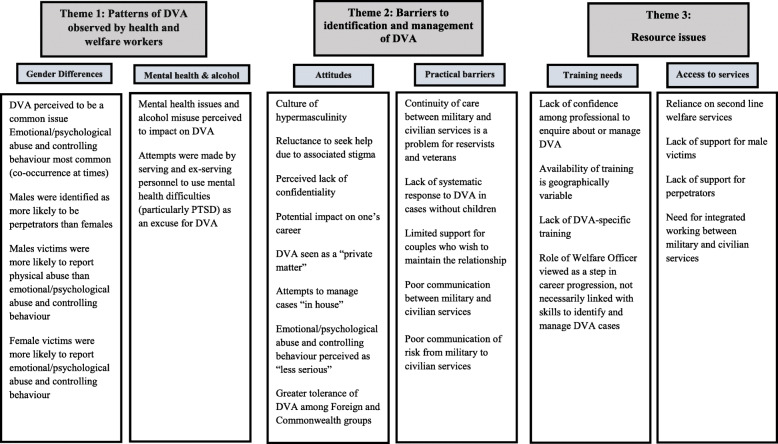
Thematic map

Figure 1 presents a visual summary of the superordinate themes and subthemes identified.

### Superordinate theme 1: patterns of DVA observed/perceived by health and welfare workers

Theme 1 explores the type of abuse most commonly reported to the interviewees (staff) by victim-survivors and perpetrators of DVA, as well as the factors that they have observed to play a role in DVA cases. Therefore, the first theme offers a description of the professionals’ experience of dealing with DVA in military communities.

Participants perceived DVA to be a common problem among military personnel (both serving personnel and veterans) but likely to be under-reported. Military Welfare/Social Worker working with serving personnel mentioned that:

*I did a straw poll of my workers back in April and sort of asked them to sort of put their hands up, anyone that had a case that had domestic abuse as a factor in it and every single one of them put their hand up.*

Similarly, a Civilian Welfare/Social Worker working with veterans noted that:

*Yes and there’s a lot of quirky things particular to the army that I have heard people talk about, because at the XX barracks, I did ask the DV question outright to the sergeant major and said ‘are you aware of any statistics around domestic violence’ and he said ‘the highest number of perpetrators in the UK have an ex service background.*

The majority of participants reported most commonly dealing with cases of emotional/psychological abuse and coercive controlling behaviour perpetrated by male serving personnel and veterans. However, some had also experience of cases of female perpetration (both by military personnel and civilian partners). Furthermore, participants observed that women who sought their support, more often cited experiences of coercive controlling behaviour, whereas male clients were more likely to report being a victim of physical abuse. Some participants observed that coercive controlling behaviour frequently accompanied other types of DVA, such as sexual and physical abuse.

The majority of participants reported that, in their experience, mental health issues (some deployment-related) and alcohol misuse played a role in many cases of DVA. This perception was shared among those who worked with serving personnel and veterans. A Civilian Welfare/Social Worker working with veterans mentioned that:

*I would say definitely that there have been post-traumatic stress disorders there as a result of the tours that they’ve done. I would say yeah most definitely mental health has been an issue linked to violence. And alcohol played a role in the domestic abuse cases. I would say definitely yes for some.*

Similarly a Civilian Welfare/Social Worker working with serving personnel mentioned that:

*We’ve had people who have got PTSD, where they’re actually sleeping with a knife under their pillow and they have a nightmare and wake up and potentially are going to attack their wife or their partner because they’ve got this flashback because of what’s going on in their heads really. So it might be that, you know, although they could be quite physically abusive to their partner, it might be that actually it’s a condition rather than, you know, that they’re a domestic abuse perpetrator.*

However, several interviewees also suggested that attempts were made by serving personnel and veterans to use mental health problems (particularly PTSD) as an excuse for DVA and expressed their frustration with this, as demonstrated by the following quotes from a Civilian Healthcare practitioner working with veterans and a Military Welfare/Social Worker, respectively.

*Perpetrators are very clever at saying they’ve got these mental health issues but actually they’re not actually diagnosed with anything, they sort of self-diagnose themself and use the military to defend their actions*.

*I do think a lot of domestic abuse has been excused for Soldiers that have come back with illnesses and injuries from tours*.

Alcohol misuse and the culture of drinking in the military were identified by participants as contributing factors in many of the cases of DVA. Both a Military Healthcare practitioner working with serving personnel and a Healthcare practitioner working with veterans reported, respectively, that:

*We are a heavy drinking culture, and alcohol and domestic violence particularly I think are very closely associated with one another. Certainly, that’s my experience.*

*It can be seen as an excuse from both parties, so like the victim might say ‘oh, they wouldn’t have done this if they weren’t drunk’, or ‘it’s only when they’re drunk that this happens’ and it can also be that people turn to drink as a way of coping.*

Lack of awareness of mental health issues was also reported as a factor. A Civilian Healthcare practitioner working with veterans noted that their clients tend not to recognise signs of anxiety or irritability. This was perceived as likely to exacerbate abusive behaviours.

*I think you’re taught not to really notice your anxiety, to just get on with things. You know, you’re focusing on the mission, the task that’s at hand, you’re not really taught to understand your feelings and sensations, you know, the ability to notice anxiety and irritability. It’s just this lack of awareness of themselves, they’ve got to focus on the mission and I think that’s another issue. I think this just exacerbates any issues really, you know, just increases the chances of something happening, so it will play a role* [referring to DVA]*.*

Thus, healthcare and welfare workers perceived DVA among the military communities to be a common issue, particularly emotional or psychological abuse and coercive controlling behaviour. Males were more often identified as the perpetrators. Practitioners observed that alcohol misuse and mental health difficulties (often deployment-related) were often contributory factors in cases they had to manage.

### Superordinate theme 2: perceived barriers to identification and management of DVA

Theme 2 explores the participants’ perceptions of the barriers which impact on the identification and management of DVA among military personnel. Two main subthemes were prominent: 1) attitudinal/knowledge-based, and 2) practical barriers. Some of these barriers were perceived to be associated with a suspected under-reporting of DVA, which in turn was reported to be a major barrier to management.

#### Attitudinal and knowledge-based barriers

The “*macho culture*” in the military was cited by many participants as a barrier to reporting abusive behaviours and help-seeking. They said that personnel are expected to be strong and able to cope with personal issues outside of their professional ‘duties’ without seeking support. This perception was shared by most of the professionals who worked with serving personnel. A Civilian Healthcare practitioner and a Military Welfare/Social Worker, both working with serving personnel, respectively reported:

*This mindset that soldiers have got to be tough and aggressive because they go into hostile and uncomfortable environments when they’re serving, I think that kind of feeds into this.*

*For them to tell their line management that they probably are a victim, I think would be almost – very seldom – I was going to say, ‘unheard of’, but it isn’t, because we have – you know, as a worker, I’ve heard of it. Yeah, I think they’d be reluctant.*

Participants working with veterans reported that the impact of the “*macho culture*” persists after they have left service and impairs recognition of behaviour as abusive and, therefore, also impedes help-seeking. A healthcare professional working with veterans reported that:

*The sort of culture of machismo that sort of exists, I don’t know if that contributes to it* (help-seeking). *We do all like to think of ourselves as being strong and fit and capable individuals, you know.*

*No matter what welfare issue we have, there’s a resistance sometimes on the ground for them to acknowledge it, because we know there’s a knock-on effect to military effectiveness if we pull people from deployment*.

Participants who worked with serving military personnel and also those who worked with veterans expressed concern that this “*macho culture*”, which emphasises male strength and bravery, has led to a lack of awareness among staff of male victimisation, while there is widespread awareness of the female victim. A professional who worked with serving personnel considered that:

*The army is a macho culture. There’s a lot of – it’s sort of – it’s all ego-driven. It’s a big meritocracy. It’s all about which man can shout the loudest, really. It’s not necessarily the best soldier that gets to the top; it’s the one who’s often shouts the loudest and is the biggest bully. Sounds awful, that, doesn’t it? But it’s probably quite true.*

Also a professional who worked with veterans noted that:

*I think – yeah, from what I’ve seen within the military, right from the word go, when I started working for them, yes, there is this definite – it’s a male-dominated environment, there’s a hierarchical structure, so there is dominance from the start, even with the rank structure.*

It was noted that these gender stereotypes can mean that appropriate action is not taken to manage female perpetrators and male victims. A Military Welfare/Social Worker working with serving personnel highlighted that:

*Our service policy uses the female tense the whole way through and signposts to places like Women’s Aid and things. I’m a bit concerned that our policy is so skewed towards a female victim because that’s just confirming that it’s not a male issue in the eyes of the organisation.*

Participants working with serving personal and also those working with veterans perceived other possible barriers to the reporting of DVA to be concerns about lack of confidentiality within military health and welfare services and fear of the impact that reporting DVA may have on an individual’s career and/or their partner’s career. A Professional who worked with serving personnel noted that:

*I think it can be quite daunting for the wives, or the husbands, but mainly it’s the wives, to just come on base – you have to get your pass, it’s kind of his or her place of work that you’re going to. I think perhaps some of them worry that their partner could maybe, would find out about it or know about it. And it’s a very small community the married patch so people see each other in the waiting area. They might know the receptionist. They might know some of the medics. I’ve had people ask me before “Can the medic see my notes?”, “Can the receptionist see my notes? Who can see my notes?” So, I think they perhaps are afraid that there’s less confidentiality.*

Participants who worked with serving personnel also perceived that cases of psychological and emotional abuse or coercive control are taken less seriously than physical abuse in the military. Indeed, many participants were of the opinion that psychological and emotional abuse is common among military personnel. However, they observed that more often than not this was not recognised as abuse by their military clients. Many expressed their view that this is likely in part due to the impact of the military setting in which individuals are exposed to aggression and hierarchy on a daily basis. A Military Welfare/Social Worker working with veterans mentioned that:

*You also get issues where somebody’s used to giving orders or they’ve had a lot of orders given to them so then with their intimate partner it’s like “you will cook the dinner by this time, it will be on the table by this time, you will have the beds made in the morning, I expect this house to be clean and tidy, of a certain standard”’, because that is drilled into them when they’re on barracks, that’s how they have to present themselves, your boots will be shining clean. So they might not even perceive this to be abusive.*

A Military Welfare/Social Worker working with serving personnel observed that:

*Coercive control is the hardest bit, you know, to get across to Units, especially if they think that someone’s a top Unit sports person or their top sniper or something. It’s very difficult to kind of get them to understand the subtleties behind it.*

Other attitudinal-based barriers included professionals’ perception of reluctance among military personnel to seek support for DVA. They described the perceived stigma associated with the reporting of abuse, such that both victims and perpetrators often present seeking help for other issues and DVA is only uncovered later in the course of the assessment or treatment. It was frequently reported that as victims and perpetrators (both serving personnel and veterans) are reluctant to disclose abuse, it is often necessary for professionals to probe before DVA is revealed.

A Military Welfare/Social Worker working with serving personnel mentioned that:

*Particularly if you’re a male victim in the military it will be embarrassment, you’re meant to be a soldier, so how can you be a victim? That will be a major factor in the cases that we’ve seen have stopped people reporting.*

Another civilian Welfare/Social Worker working with serving personnel observed that:

*I’ve certainly seen a few cases where the initial referral has been about something to do with mental health and then it actually transpires that in actual fact it was DVA perpetrators and victims.*

Similarly, a Healthcare practitioner working with veterans shared that military personnel are likely to be reluctant to ask for help while serving, due to fearing the impact on their careers. Thus, from their experience, military personnel tend to reach out for support from non-military services.

*I would say when they are still serving, they are more reluctant to disclose* [DVA]. *There is fear of that information getting back them. So, definitely more reluctance when serving, they might still be worried about confidentiality.*

Healthcare and welfare staff working with serving personnel raised concerns about identifying DVA among Foreign and Commonwealth military groups. Participants perceived that they display higher levels of tolerance of DVA. For example, our participants reported that Fijian and African families and the Gurkha communities they worked with have been actively discouraged from reporting of DVA by those in their senior ranks. A Military Leader reported that different culture and social norms, fear of being deported to their home countries, as well as lack of alternative recourses might also contribute to non-reporting if DVA.

*In particular for those who are foreign commonwealth, there’s the added issues of if they don’t have indefinite leave to remain here and no recourse to public funds and the only alternative is that if they leave their husband they have to return to the country of origin. The country of origin’s cultural norms might be that they’re then outcast from the family, put into poverty and poverty in the true sense of the word as opposed to you know, just living in our sense of poverty. So that would encourage women to stay in abusive relationships because actually it’s better than the alternative.*

Several participants working with serving military personnel reported that attempts are made to manage cases of DVA “*in-house*”, in order to protect serving personnel perpetrators if they are needed for operational deployment. They reported that this can obstruct integration between civilian and military services and impede the effective management of DVA and communication of risk. Military Welfare/Social Workers working with military personnel reported that:

*There’s a lack of understanding in the military. Because we’ve got to be fully effective, I think if people are due to be deployed and we suddenly flag up that there’s an issue, which we would do if there was children, if there was a risk, if there was children protection/safeguarding, it may be that we would have to highlight that the serving person could not go away because of the issues that we were dealing with.*

Another Military Welfare/Social Worker working with serving military personnel observed the following:

*What happens is if a soldier was abusive to his partner and everybody heard about it, he’s put in the can or whatever they call it, he’s put in military prison. Everything’s in-house. And ironically that prison is on the garrison that you’re living in with your children.*

A number of participants reflected on attitudes among some within the military that, as an employer, it should not be getting involved in DVA cases. A Military Healthcare professional working with serving military personnel mentioned that:

*There is a cultural blind eye in the Military to it I think, people in Units may know it’s going on, but they don’t intervene because they feel it’s a private matter.*

In fact, they reported some cases where their clients’ relational difficulties were perceived by the military hierarchy as a ‘*private matter’*.

#### Practical barriers

In addition to the attitudinal and knowledge-based barriers to identification and management described above, participants reflected on practical barriers. Many participants, both those working with serving military personnel and also those working with veterans, mentioned that, in the absence of the involvement of children, cases of DVA in the military were less likely to be acted on. A Military Healthcare professional working with serving personnel mentioned that:

*Where there are couples without children I think Unit Welfare Staff are more likely to pass it off as they’re just having an argument especially for those who are cohabiting in single living accommodation you know in the big blocks*.

In contrast, it was reported that when children are in the household, DVA cases are likely to be automatically referred to Military Welfare Services or community social services. The referral pathway for cases where children are not involved seems to be less clear. Similarly, participants felt that it was easier to manage cases of physical abuse, as the decision to breach confidentiality is based on more objective evidence warranting a referral to Social Services or the Police.

A Military Welfare/Social Worker working with serving personnel reported that:

*When you think of where child protection is now, you know, and people won’t tolerate children being abused, you know, much more likely to report it and the statutory requirements on people to report. Obviously with domestic abuse it’s kind of a little bit behind with that.*

Similarly, a Healthcare professional working with veterans mentioned that:

*Nearly every alcohol client that I was referred there were domestic violence issues as well. So the children, nearly all of the families that I work with, the children were put on child protection plans for a dual issue, dual adult issues, they were often, you know, they were put under the category of neglect, let’s say, the children, but the issues for the adults were alcohol and domestic violence. I guess the policy is more straightforward when children are involved too.*

Participants (mainly those who worked with serving personnel) observed that within the military, competing priorities can be problematic for identification and management of DVA. Indeed, several interviewees described the prioritisation of military operational effectiveness over family difficulties. A Civilian Welfare/Social Worker working with serving personnel reported that:

*It’s about operational effectiveness, it doesn’t matter what your organisational output is, but if your whole organisation is geared towards that, and it is about being successful, and achieving a mission, you know, by showing weakness you’re – yeah, so, yeah, so some of the cases I saw it felt like – they were not supporting the team’s output, I guess* (by focusing on the family difficulties). *You’re kind of letting your team down*.

Participants who worked with serving personnel also highlighted that frequent relocations and transition out of the military were perceived as factors which can disrupt contact with military health and welfare services and impair identification and management of DVA. It was suggested that it may be particularly difficult to identify and manage DVA among reservists and their families due to the irregular contact they have with their unit. A Military Welfare/Social Worker working with serving personnel mentioned that:

*The challenge of getting a message out to the army reserve is greater than giving it to a regular unit, just by the nature of the dispersed footprint of army reservists, and actually being able to connect with them all and for the army reservist’s family to perhaps even more so know that the army has an organisation that will support them, even though they’re the spouse of an army reservist*.

Difficulties in management were partly attributed to poor communication between different military services, as well as between military and civilian services. In particular, a number of participants highlighted instances where a lack of communication of risk by health and welfare staff had contributed to a perpetrator engaging in further abuse in a new relationship after leaving the military having previously been identified as a perpetrator while serving. A mental health practitioner working with veterans said the following:

*I’ve had a number of patients who have told me they perpetrated DVA against a previous partner while serving, which was reported to and ‘managed’ by the military. That relationship has perhaps ended, the person leaves the military and there has been no communication with his GP or community services that they are a potential risk to future partners.*

It was of note that a professional working with veterans who have experienced abuse felt that the referral process and communication between different agencies can be problematic.

*t’s a slightly odd situation isn’t it because whereas normally you have your records of practice, there is this split isn’t there, you know, so the serving personnel is under the med centre, but then this is not communicated when they leave. So that makes it slightly more tricky sometimes for* [DVA] *to become apparent.*

Thus, professionals working with both serving military personnel and veterans perceived the lack of communication between services and agencies to impact on the identification and management of DVA negatively.

### Superordinate theme 3: resource issues

The third theme offers an overview of resources issues perceived by the professionals. Two subthemes were identified: 1) training needs, and 2) access to services.

#### Training needs

While there were mixed views about whether DVA is taken seriously in the military, it was clear that participants felt that awareness and management of DVA are improving, and that there were more opportunities for training.

A Military Leader reported:

*We have some external police talkers as well and they had a really good package which included a few videos and real life 999 calls and that kind of stuff which, you know, certainly from my perspective, is what’s really stuck with me from that training, you know, in terms of how severe this can be if it’s not -, if we don’t support those that are going through this.*

Some participants working with military personal and those working with veterans have also highlighted that they felt that public visibility of information on DVA and support services has improved, and this has contributed to an increased awareness among military communities. A Military Welfare/Social Worker working with serving personnel reported that:

*The Welfare Services have got much, much better at advertising their services and getting involved with the families getting the trust of the families, you know, people perhaps do feel more able to come forward.*

A Civilian Healthcare professional working with veterans noted that:

*The Army Welfare Service has been doing multi-agency conferences and training. So I would say from that I think that it’s become more aware of domestic abuse and that they want, you know, their protocols and procedures to be good and they want to, you know, they want to help I think.*

Furthermore, all participants in Leadership roles expressed their motivation to raise awareness and improve practice, as can be demonstrated by the following quote from a Military Leader.

*I think as an organisation, we are so, so keen to get rid of some of our ghosts, you know, in terms of things like bullying and not being inclusive, and you know, this macho culture, that the Chain of Command absolutely is crystal clear that this kind of behaviour is not acceptable. And you know, it needs to be dealt with. I’m certainly absolutely crystal clear on* that.

However, others professionals such as Military healthcare professionals, particularly those who worked with serving personnel, were not observing the impact of this top down enthusiasm. A Military healthcare professional working with serving personnel reported:

*They want to be seen to be taking it seriously, but if they were really taking it seriously they would be looking into what can we do to address it, educational programmes. None of this as far as I’m aware exists. Certainly, I’ve not seen any posters for domestic abuse whereas I’ve seen lots of posters for other things.*

Professionals working with veterans described that training to effectively work with perpetrators of DVA is lacking. As demonstrated by the quotes below from a mental health professional working with veterans:

*I think where the training is not detailed enough is how you deal with perpetrators. I have gone on perpetrators training but I would say that still is really, yeah, I think really quite hard to deal with.*

*I think there needs to be more training around DV and understanding of what is it. Because I do think perpetrators fall into different categories, I don’t think there’s just a, you know, in the DV field perpetrator is often just seen as a bad person but it isn’t as simple as that, it really isn’t. So I suppose I’m coming from a different stance. It’s just not straightforward.*

Despite the mixed views, the need for greater awareness and training in the identification and management of DVA was frequently reported by professionals working with both serving personnel and veterans. Some first line welfare and healthcare staff interviewed reported that they had not received any specific DVA training, and had gained their knowledge through more generic safeguarding training or learning from others. A Military Welfare/Social Worker working with serving personnel reported that:

*The training needs to be rolled out. But it needs to be training that’s meaningful. Sitting in front of a computer doing an online awareness course that you tick a box at the end to say you’ve done it isn’t enough. It needs to be real kind of, it needs to be interactive training where people are actually able to hear and be challenged on their thinking you know.*

Similarly, professionals working with veterans perceived that not all members of their teams were skilled to identify and respond to DVA cases, particularly where violence seems to be hidden.

*What some of my colleagues are doing is because the person says ‘oh she said I was violent but none of it’s true’, it seems to end there, they’re not inquisitive and then even if the client then goes on and gives a description of a very violent incident, they seem to dismiss that.*

*I still think there could be more training. To be honest only recently our local authority have started doing training. You know we had to actually find training ourselves from an outside agency who were really, really good. But yeah I think there needs to be more of that to be honest.*

Consequently, levels of confidence in identifying DVA, asking the right questions (particularly of perpetrators), dealing with cases and awareness of DVA management policies varied widely among participants who worked with both serving military personnel and veterans. A Military Welfare/Social Worker working with serving personnel reported that:

*I think everyone needs to be asking about it routinely. But it’s about having the confidence to ask the right questions and to, as I said, you’d go* via *the back door a little bit. Yeah, and I think we need more training around that. It’s not easy. I think there’s a lot of concern about ‘Oh if I ask that maybe it’s going to make it worse’.*

Another Military Healthcare practitioner working with serving personnel observed that:

*I think there’s a huge barrier in terms of asking perpetrators. I think it’s much easier to ask a victim, someone that you expect, you know, suspect is a victim, so much easier to ask about the victim. It’s so much harder to actually get a perpetrator to talk about what he’s done.*

Similarly, a Civilian Healthcare professional working with veterans noted that:

*Asking about domestic abuse routinely is key. I mean as part of the risk assessment, we should be doing that as part of the risk assessment, yeah, comprehensive assessments.*

It was frequently noted that the position of the Unit Welfare Officer is often achieved as part of a promotion pathway rather than being based on welfare experience or interest. Participants working with serving military personnel commented that this can impact on the person’s suitability for the role, their approachability and hence their ability to identify and manage cases of DVA, as is demonstrated by the following quote from a Military Welfare/Social Worker.

*The thing is with unit welfare officers, you know, it’s not the top ten of everybody’s job. Now, I asked to come and do this job because it’s a job that I wanted to do, whereas a lot of people, they are promoted from soldier to officer and the first job they get is unit welfare officer. Now, some people wanted it and are like, ‘Yeah, I’m happy to do that’, and other people just see it as a rite of passage.*

Although the burden of identification of DVA among serving personnel falls to military healthcare and welfare workers, a recurrent theme was a lack of confidence in dealing with DVA among first-line staff, leading to considerable reliance (some felt this was over-reliance) on second line welfare services (i.e., the AWS and SSAFA) for the management of DVA, as mentioned by a Military Welfare/Social Worker.

*What you can tell is I’m very reliant on having [second line welfare services] workers, and when they are not here then you feel a little bit more vulnerable because I’m taking more responsibility on for stuff that I’m maybe not as well-trained in.*

Similarly, some participants working with veterans reported feeling ill-equipped to deal with DVA and needing to rely on community social services or specialist DVA services.

*We’ve got protocols on safeguarding adults and safeguarding children, but not a specific protocol on domestic violence.*

#### Service level needs

Participants working across all branches of the military and those working with veterans reflected on the importance of having strong working relationships between first line services (welfare and health) and second line welfare services, social services and specialist DVA support services. Some participants working with veterans expressed the view that veterans would benefit from support services with specific knowledge of the military experience, rather than generic civilian services.

*We need to make sure we do integrate their military background. I’m sure that some veterans are controlling and coercive and yeah just not very pleasant, other veterans might be strangling their partner in the middle of the night because they’re experiencing symptoms, but it wasn’t their intention to harm them, but that partner’s nevertheless in danger. And you’ve got another veteran who is disciplining the children or expecting that partners are doing as they’re told, bringing military culture into a family environment. So this has to be considered by the professionals.*

Some were aware of perpetrator programmes offered by RESPECT (a UK charity that offers helpline support for male and female perpetrators of domestic violence and abuse), but reported reluctance among veterans to self-refer to ‘civilian’ services and very long waiting times. A Civilian Healthcare worker working with veterans mentioned that:

*Right, for victims, I know of masses of services, but they’re all for everybody, they’re generic not based on any sort of military experience, and have long waiting lists. For the armed forces, there is X service. But my understanding is that either the victim or perpetrator has to be currently in the services*

Furthermore, Professionals working with veterans noted that the pathways to support perpetrators are unclear, and that they were more aware of agencies to support victims/survivors of DVA than perpetrators.

*For the perpetrators there never has been enough support, so that’s not just for the armed forces. I only know of DVIP and what’s the other one, Respect, yeah there’s hardly anything in London, nothing for perpetrators. Or if they go through the probation route, sometimes probation are doing their own -, and of course they would have had to have been charged with an offence of domestic violence, so and then they’re put on some perpetrator courses.*

The majority of participants working with serving personnel and veterans cited the importance of integrated working between military and civilian services in order to better manage DVA and the associated risk. Participants working with veterans observed that the structure of the military provides containment for individuals, especially those with challenging early lives, and the destabilising impact of leaving the military can increase the risk of aggression in general and this can manifest as DVA in the home environment when they leave. A Civilian Healthcare professional working with veterans observed:

*The military gives the structure and containment that many have not had in their early lives. I think, you know, I would say that many of those I see in my clinics have joined the military to escape difficulties in their early civilian lives. You know, and they find they can channel their anger and aggression into a useful role. But when they leave, eh, when they no longer have the military regime, the purpose that the military provided, and the containment is gone, that risk of aggression can increase and for many that plays out at home.*

The issue of lack of preparedness for the transition from military to civilian life was raised. A Civilian Healthcare professional working with veterans reported that:

*Veterans are different from anyone else. I just think that it needs to be more understood, the nature of some of the DV. I do think that they have all this you know, in terms of like military culture and making the adjustment to civilian life and the hyper-arousal symptoms you know it does make them more volatile. They make great security guards but they’re not taught that they don’t need to do it any more in civilian life, it’s all that that predisposes them to DV.*

The importance of providing effective continuity of health and welfare support for individuals transitioning out of the military was highlighted. A Healthcare practitioner working with veterans mentioned:

*I’ve had a number of patients who have told me they perpetrated DVA against a previous partner while serving, which was reported to and ‘managed’ by the military. That relationship has perhaps ended, the person leaves the military and there has been no communication with his GP or community services that they are a potential risk to future partners.*

Several participants considered that integrated working between military and civilian services may be particularly relevant for reservists and civilian spouses, as participants felt they are more likely to utilise civilian rather than military support. Indeed, many participants discussed how the military do not provide, in general, support to spouses/families of reservists. A Healthcare practitioner working with serving military personnel mentioned:

*Because the reservist will only have irregular contact with their unit potentially, at most, once a week, generally a lot, lot less. Those families particularly wouldn’t necessarily know who to get to in the military to seek support. On the other hand they potentially have a sort of stronger support network locally hopefully.*

A gender disparity was observed in the provision of support for male and female victims and perpetrators. Participants observed that gender stereotypes in the military mean that appropriate support is less often offered to male victims compared to females. Conversely, some observed that action is often not taken to manage female perpetrators in the same way as it is with males. A Welfare/Social Worker working with serving military personnel noted that:

*It seems to be the assumption that the violence is being perpetrated against the female but actually, like I say that I had cases, it’s been the other way round quite interestingly. I’m a bit concerned that our policy is so skewed towards a female victim because that’s just, in many ways, for me confirming that it’s not a male issue in the eyes of the organisation.*

A general lack of support for perpetrators was frequently mentioned. Only a small number of participants were aware that, at the time of the research interviews, the AWS were piloting a military perpetrator programme at three sites in England, while most participants working with both serving personnel and veterans reported that they were not aware of a perpetrator programme. Consequently, participants felt that perpetrators are often incorrectly referred to mental health services. Healthcare staff also mentioned feeling ill-equipped to manage perpetrators. Moreover, the absence of protocols for the management of DVA within couples in order to work with the both partners together was reported. Many participants described how often victims do not actually wish to leave the relationship and some couples would prefer to work through their difficulties with support and joint working, but military services seemed reluctant to work with the civilian spouse. To compound this, it was also observed that there is a lack of community services capable of working with victims and perpetrators together, as mentioned by a Civilian Welfare/Social Worker working with serving personnel noted that:

*I honestly can say in all the years I’ve done this job that the amount of soldiers that have said “I know it’s not good. I want it to be different” and we’ve not been able to provide them with the support that they’ve needed to make the changes, and that’s really, really difficult.*

Finally, participants reflected on the beneficial services and resources that are in place for the management of DVA in the military, such as free relationship counselling provided by RELATE (a UK charity that offers counselling and mediation for couples and families) and a tri-service confidential phone line providing a supportive listening and signposting service. Several participants also praised SSAFA and the AWS for the helpful support they provide. Some serving healthcare and welfare workers acknowledged that the military have a range of branch-specific services to which they can signpost personnel, including post-deployment reintegration support for families, access to a perpetrator course (if located within the catchment area for a pilot course) and DVA victim-survivors counselling. It was felt that access to confidential services, such as the availability of padres as part of the welfare team, is important. However, it was apparent from the responses of participants in different parts of the UK that the availability of services varied by geography, as mentioned by a Civilian Healthcare practitioner working with serving personnel: *In* [geographical location] *they had domestic abuse awareness days regularly, but at the station where I am now DVA does not seem to be a priority*.

## Discussion

The findings of this study shed light on the experiences of healthcare and welfare workers in identifying and responding to DVA among military communities. DVA was perceived as a common issue among serving personnel and veterans. According to the majority of our participants, psychological and emotional abuse and coercive controlling behaviour were most frequently reported by their clients, consistent with findings from quantitative studies on DVA in the military [5, 47]. Mental health problems and the culture of drinking in the military were seen as important factors in some cases of abusive behaviour, also in keeping with international research [48]. Furthermore, the professionals interviewed observed that females who sought help were more likely to report being victims of coercive controlling behaviour, and males were more likely to report perpetrator or experiencing physical abuse. It is important to note that this is based on the professionals’ perceptions. Nevertheless, this may reflect a lack of reporting of emotional abuse or coercive controlling by male victims, due to shame or embarrassment. Additionally, perpetrators may not recognise this behaviour as abusive. Research among ex-military male perpetrators has similarly reported that they do not perceive controlling behaviour to be abusive [49]. However, it is also possible that there are gendered differences in help-seeking, which may be greater in military populations [50–52].

Most participants believed that the full extent of the problem of DVA in the military remains hidden due to under-reporting. They reflected on attitudinal or knowledge-based and practical barriers to the identification and management of DVA among military personnel, including: the stigma associated with reporting abuse and seeking help (echoing previous research within military personnel [53, 54]; the ‘*macho culture’* and focus on self-reliance in the military which can lead to the perception of help-seeking as a sign of weakness [55]; the perceived lack of confidentiality within Armed Forces welfare services and fears of the potential impact on careers or that of their partners. Other research has shown that this stigmatising association between help-seeking and weakness often persists even after individuals have left service [51]. Stigmatising beliefs about help-seeking among serving military personnel and perceived negative impact on one’s career have been shown to predict dropout from mental health treatment [56] and such concerns are just as relevant in help-seeking for DVA.

Participants’ narratives also reflected a perception of a lack of recognition of male DVA victimisation in the military and the lack of support available for male victims. Previous research has suggested that there is a gender-bias in the military which shapes responses to abuse [57], and male DVA victims in the general population have spoken of negative experiences of help-seeking, such as being ridiculed by the police [58]. It is however also known that men who perceive themselves as victims may in fact be the perpetrator of more severe abuse [59]. It is unlikely that currently, welfare workers would have the skills to establish this and likely that our findings suggest that male victims and perpetrators are at risk of poorer care.

Considering that DVA victimisation has been found to be prevalent among male military personnel internationally [24, 25, 47, 60–62], increased awareness of male victimisation in the military is needed. Furthermore, participants perceived that healthcare and welfare staff are often ill-equipped to manage cases of psychological abuse or coercive control and that those forms of abuse are likely to be taken less seriously than physical abuse. Greater awareness is needed of the spectrum of DVA across all genders as well as confidence in managing DVA which does not involve just physical abuse.

It was observed that sometimes attempts are made to manage DVA “*in-house*”. Previous research has highlighted that although military welfare services exist to support military families, both first line and second line services are ultimately motivated by the needs of the military institution [53]. For instance, the AWS Mission Statement explicitly states their aim to provide a confidential support service in order to maximise effectiveness of personnel [57]. Gray [53], in her qualitative exploration of the response to DVA in the UK military, identified that emotional abuse lacked relevance to operational effectiveness, meaning it was a “*private matter*” and the military should not get involved. This was a theme in our study also. Given the growing awareness of the impact of military service on the families of personnel, such conflicts in priorities must be considered by the military and strategies to overcome them developed.

Participants in our study discussed perceptions of a greater tolerance of DVA within some Foreign and Commonwealth communities. Attitudes to DVA in different societal contexts are known to vary and impact on prevalence and a growing awareness of DVA among Fijian personnel has been reported in previous research within the British military [57]. The circumstances of Foreign and Commonwealth spouses (e.g., their reliance on their marriage for the right to remain in the country and on their spouse financially, and their isolation from their own communities), and the power imbalance within these couples, may impair their ability to seek help. Those who do not have a ‘Right to Remain’ may not report abuse due to fear of being deported. Such vulnerable groups must be better supported, informed of their rights, and provided with opportunities to seek help [63].

Healthcare professionals in the general population have reported that lack of continuity of care is a barrier to DVA screening and response, as patients cannot build trusting relationships with care providers if they see a new professional at each visit [64]. Our participants affirmed this citing the frequent relocation of military families as a barrier to DVA support. Participants also felt that DVA is less easily identified among reservists due to the irregular contact they have with their Unit. This is problematic, considering that UK reservists deployed to the 2003 Iraq War have reported lower marital satisfaction and increased problems adjusting to homecoming than Regular personnel [65]. The lack of clear protocols for responding to DVA was also highlighted as a barrier, particularly where there were no children in the household, leading to often inconsistent and unsystematic responses [40, 66, 67].

Participants emphasised the current lack of specialist training in identifying and responding to DVA, which should include how to ask about DVA, as well as resources to support any service wide efforts to improve support. Lack of confidence in managing DVA is known to be a significant barrier to DVA screening and management in many healthcare settings [18, 34, 40, 66, 68] and not surprisingly it was also reported in our study [64]. DVA is frequently overlooked by healthcare professionals because abuse is not something that people are open about and “*it takes time and meandering to get it out*” [64]. The National Institute for Health and Care Excellence (NICE) recommendations highlight the need for training which includes how to enquire about DVA, and safely respond, with routine enquiry implemented in people attending all health and social care settings [31].

The burden of DVA identification falls to healthcare and first line welfare workers so it is imperative that they feel confident in screening procedures and are knowledgeable about second line/specialist services to refer to. It has been highlighted that GPs and mental health professionals are well placed to ask about abuse because DVA victims are more likely to consult health services than any other organisation [31, 69–71]. Outside of the military, due to ever increasing demands and time constraints, it has been proposed that a healthcare professional’s role should be to identify abuse and refer on to DVA advocacy/specialist services, as opposed to becoming involved in the ongoing management of DVA [31, 69, 70, 72–74]. It should be considered whether or not, in military settings, it would also be helpful to differentiate between the predominant responsibilities of healthcare/first-line welfare workers (identification and signposting) compared to second line/specialist services (DVA management) and to focus training accordingly.

Research in healthcare settings has shown the importance of providing a clear referral pathway to a DVA advocate/specialist services to encourage disclosures and provide effective support [70, 75, 76]. A cluster randomized controlled trial (RCT) in primary care found that advocate educators integrated into practices significantly increased identifications and referrals [77]. Evaluation of a similar intervention in military settings would be very helpful in establishing whether there may be a beneficial role for DVA advocates within military healthcare settings to improve identification and the referral pathway to specialist support.

A particular lack of support available for perpetrators was evident from our interviews. This is a challenge for the field of DVA more broadly, and more research is needed on the most effective interventions for perpetrators [31] and any modifications needed for military personnel, considering the unique challenges they face (e.g., military training, combat exposure). A cognitive behavioural trauma-informed intervention for male military perpetrators in the US has shown promising reductions in abusive behaviours and could be trialled in the UK [78].

An identified barrier to the reporting of DVA is that many victims do not wish to leave the relationship, often owing to the perceived threat of material losses [44, 46]. This may be intensified for the civilian spouses of military personnel, as the “*military package*” may include housing and children's education fees, which may be lost if they leave the relationship. Welfare workers felt that couples interventions may be helpful. However, current evidence on couple interventions suggests that they can result in negative outcomes due to the power dynamic that is hidden from the therapist, particularly where there is coercive control. A CBT based couples program for military partner emotional and physical abuse in the US has shown reductions in abuse in a randomised controlled trial [79]. Such a program could be trialled in the UK. For now, NICE recommend assessment and interventions that can be provided to both members of the couple individually in addition to any joint sessions [31].

The importance of strong working relationships between services for the effective management of DVA was emphasised in this study and in previous research in both civilian and military populations [44, 46]. Our participants felt that more integrated working between civilian and military services is particularly important for reservists, veterans and civilian spouse victims. Civilian partners in the UK Armed Forces have reported feeling that the military bubble is not an appropriate place for them to access support [57]. However, many were also not aware of how to access civilian services and reported that they were not advertised in the same way as military resources [57]. Whether in a generic or military-specific service, an understanding of the unique military culture and experiences and how they may impact on risk of DVA is important when providing effective and person-centred support to military families [79].

### Strengths and limitations

This is the first study to examine the experiences of healthcare and welfare workers in managing DVA among serving personnel and veterans in the UK. The qualitative approach enabled us to provide an in-depth description of the experiences of these practitioners. The diversity of our participants in different settings and regions in the UK, as well as with different ranks and leadership roles, meant that we could not draw comparisons between all the different potential subgroups. The total sample size of professionals was adequate for this qualitative research [80] as data saturation was achieved. However, differences across the branches of service exist, such as variation in the set-up of welfare services and housing circumstances, which are likely to influence the experience of DVA, but examination of such differences was beyond the scope of our study. Further research into the impact that branch of service has on the management of DVA in the UK Armed Forces would be valuable.

### Future directions

There have been recent advances in the UK military’s approach to DVA in terms of improved awareness, training and management. A commitment to continuing to progress in this direction was evident among our participants in leadership roles. However, there is more work to be done. At an individual level, increased awareness of the spectrum of DVA, particularly of emotional and psychological abuse and coercive control and male victimisation, is necessary to remove barriers to adequate support for military families. At an organisational level, standardised procedures for DVA management and systematic training are required to promote a consistent and appropriate response to DVA. There is a particular training need among healthcare and first-line welfare staff, who are largely relied upon to identify cases of DVA.

Military personnel in leaderships roles must continue their efforts to change the prevailing culture toward one in which domestic abuse is not tolerated, normalised or considered beyond the remit of the military, and in which helpseeking is encouraged and supported. Employing independent DVA advocates within military and civilian healthcare settings may be useful in improving DVA management and access to specialist support. Improved integrated working between civilian and military services may increase the accessibility of support and counteract concerns around the confidentiality of welfare services and impact on career. Better communication between services will also help to ensure that adequate care is provided to civilian partners and those transitioning out of the military.

## Conclusions

Under-reporting is an impediment to the management of DVA among serving military personnel, veterans and their families. Aspects of military service, in terms of its institutional culture, attitudes and practices, are observed to present additional barriers to help-seeking which must be addressed. Effective responses to DVA by military healthcare and welfare services are dependent on a culture that shows awareness and competence at both individual and organisational levels.

## Supplementary information

**Additional file 1.**

## Data Availability

The datasets used during the current study are available from the corresponding author on reasonable request.

## Abbreviations

DVA: Domestic violence and abuse / Intimate partner violence
PTSD: Post-traumatic stress disorder
NHS England: National Health Service England
RAF: Royal Air Force
AWS: Army Welfare Service
RNRM: Royal Navy Royal Marines
SSAFA: Soldiers, Sailors, Airmen and Families Association
VHA: Veterans Health Administration
