# Supplementary material for: The experience of health and welfare workers in identifying and responding to domestic abuse among military personnel in the UK
Source: BMC Health Serv Res. 2020 Oct 15;20:947. doi: 10.1186/s12913-020-05672-x (PMC7559780; doi:10.1186/s12913-020-05672-x)
Supplement: Supplementary file 1 — Additional file 1. [file 12913_2020_5672_MOESM1_ESM.docx]

**Topic Guide**

1. Awareness of problem of DVA in military.
2. Insight into problems that can lead to DVA.
3. Insight into problems with identification and response to DVA in military
4. Barriers to reporting DVA perpetration or victimisation?
5. Awareness of protocol for reporting/managing DVA.
6. Support services available.
7. Recent changes in attitude to DVA in military?
8. Recent changes in how military respond to DVA in military?
9. What could be improved?
